# Bladder Pain Syndrome With Repeated Bladder Hydrodistention—A Case of Functional Somatic Syndrome Considered in Relation to Alexithymia

**DOI:** 10.1002/iju5.70007

**Published:** 2025-02-24

**Authors:** Naoki Wada, Tsubasa Hatakeyama, Taichiro Ishimaru, Ryoken Tsunekawa, Kotona Miyauchi, Daiki Kikuchi, Takeya Kitta, Masaki Watanabe

**Affiliations:** ^1^ Department of Renal and Urologic Surgery Asahikawa Medical University Asahikawa Japan; ^2^ Department of Urology Kitasaito Hospital Asahikawa Japan

**Keywords:** alexithymia, bladder pain syndrome, hydrodistention

## Abstract

**Introduction:**

We present a patient with bladder pain syndrome (BPS) who underwent repeated bladder hydrodistentions.

**Case Presentation:**

A female patient visited our department because of refractory bladder pain. She was diagnosed with BPS; she had only mucosal bleeding after distention. Her bladder pain improved after hydrodistention; however, the symptoms flared up within a few months. She also consulted with the palliative care department and was diagnosed with chronic pain associated with alexithymia. Various drugs were administered; however, none were effective or continued because of side effects. It was also challenging for her to embrace introspective counseling. Ultimately, along with her strong desire, the hydrodistention continued every few months. Her bladder capacity was approximately 200 mL.

**Conclusion:**

BPS with uncontrolled bladder pain may be a functional somatic syndrome associated with alexithymia, and interventions such as psychosomatic medicine could be necessary from the early stage.

**Abbreviations**
BPS: bladder pain syndrome
FSS: functional somatic syndrome
IC: interstitial cystitis
LUTS: lower urinary tract symptoms

**Summary**

- In bladder pain syndrome (BPS) with uncontrolled bladder pain, functional somatic syndrome (FSS) may occur.
- Early interventions by a psychosomatic physician or other healthcare provider may be necessary without blindly resorting to surgery.

## 1 Introduction

BPS is a chronic condition characterized by pelvic pain and lower urinary tract symptoms (LUTS), without Hunner lesions characteristic of interstitial cystitis (IC) [1]. In BPS, bladder hydrodistention has shown some efficacy; however, no highly effective treatment method has been established.

Alexithymia is a personality trait characterized by difficulties in identifying and describing emotions [2] and is considered one of the background factors for FSS, including BPS, irritable bowel syndrome, tension headaches, temporomandibular disorder, premenstrual syndrome, or fibromyalgia. Herein, we present the case of a patient with BPS and alexithymia who underwent repeated bladder hydrodistentions.

## 2 Case Report

**FIGURE 1:**
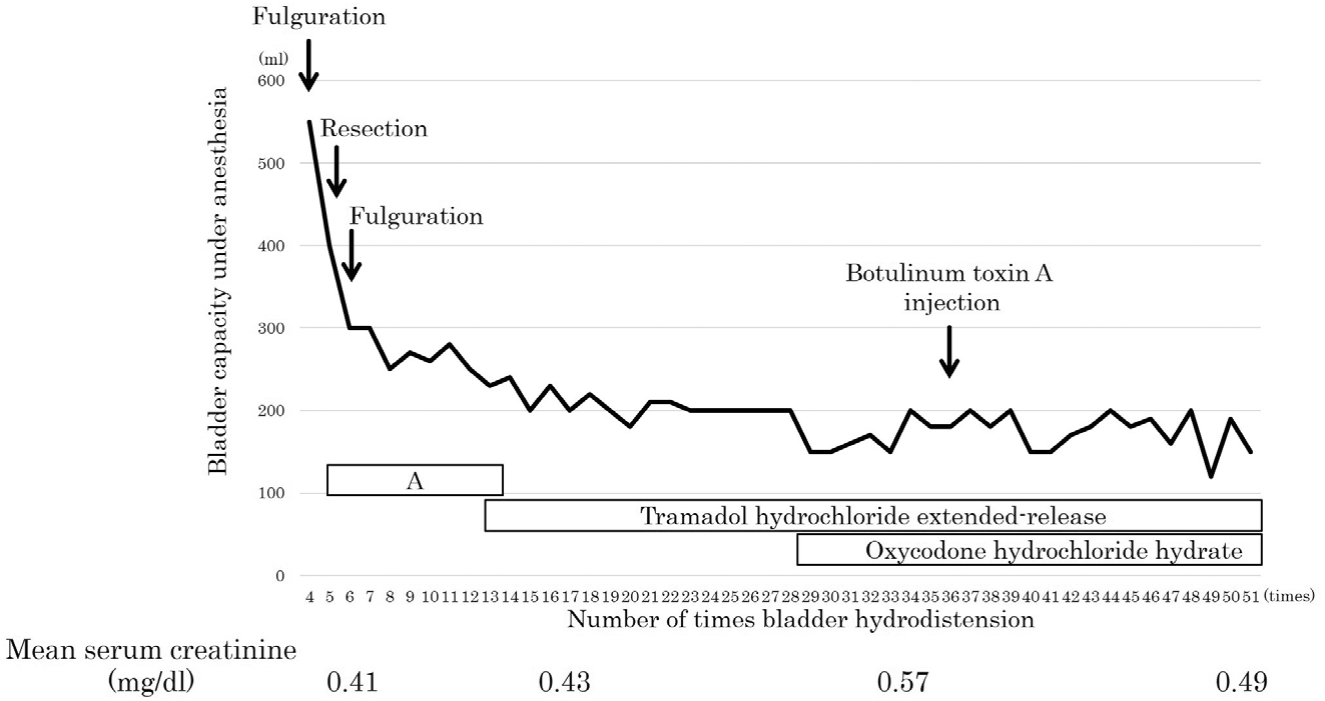
Changes in bladder capacity during bladder hydrodistention under anesthesia and serum creatinine level. Since hydrodistention procedures up to the third time were performed at the previous hospital, data from the fourth time onward are presented. Because of the lack of improvement in bladder pain and concern of missing Hunner lesions, fulguration or resection of the mucosal hemorrhage area was performed during several initial hydrodistentions. During period A, nonsteroidal anti‐inflammatory drugs, pregabalin, sodium valproate, carbamazepine, mirtazapine, duloxetine hydrochloride, mirogabalin besilate were administered, but none of the drugs were effective. In the last 7 years, the patient has been prescribed mainly tramadol hydrochloride, with additional oxycodone hydrochloride being prescribed for the last 4 years. However, these drugs alone do not control bladder pain. Urinary frequency is increasing in daily life along with decreased bladder capacity under anesthesia. Every hydrodistension significantly reduces bladder pain, but after a few months, the pain flares up to maximum. Thus, for the past few years, the patient has planned to do hydrodistension before the pain flares up.

A female patient in her 30s who had urinary frequency and bladder pain was diagnosed with BPS by a previous physician 3 years before she visited our department. She had undergone bladder hydrodistention three times in those 3 years. She was referred to our department because of refractory urinary frequency and bladder pain. She had undergone a transvaginal hysterectomy for uterine adenomyosis and ablation for paroxysmal supraventricular tachycardia 4 months before her referral. Cystoscopic evaluation did not detect Hunner lesions or other intravesical abnormalities, only mucosal bleeding after distention. Hydrodistension is usually done with an 80 cm water column and holding for 5 min. Her bladder pain improved after hydrodistention under anesthesia; however, the symptoms flared up within a few months. Frequency–volume charts showed average and maximum volumes of 50 and 130 mL, respectively. Abdominal computed tomography and spinal magnetic resonance imaging findings were normal. Nonsteroidal anti‐inflammatory drugs, pregabalin, sodium valproate, carbamazepine, mirtazapine, duloxetine hydrochloride, mirogabalin besilate were administered for bladder pain but were ineffective or discontinued because of side effects. Because of the unimproved bladder pain and concern of missing Hunner lesions, fulguration or resection of the mucosal hemorrhage area was performed during several initial hydrodistentions (Figure 1).

The patient also consulted with the palliative care department. Since the patient strongly refused to see psychiatrists, a palliative care physician with training in psychiatry was consulted for pain management as well as psychological intervention. During the interview with the palliative care physician, the patient was diagnosed with alexithymia because she had physical sensations that even physicians do not understand or it was difficult for her to find the right words for her feeling. She had taken various drugs, such as morphine, lorazepam, and etizolam, which were ineffective. She found it challenging to accept introspective counseling because she was unable to acknowledge that her symptoms might have a psychological origin. In the end, along with her strong desire, the hydrodistention continued every few months. During the 10 years of treatment, she has suffered from tension headaches and temporomandibular disorder. Over the past decade (> 50 hydrodistentions), her bladder capacity under anesthesia has decreased from 550 mL to 200 mL; however, no evidence of hydronephrosis or renal dysfunction was found (Figure 1).

## 3 Discussion

Herein, we presented a patient with BPS associated with alexithymia who had undergone > 50 bladder hydrodistentions to date. Looking back on the course of the case, fulguration or resection of the bladder mucosa may have undeniably caused a decrease in bladder capacity despite the absence of Hunner lesions. If the BPS in this case had been diagnosed early as an aspect of FSS associated with alexithymia, psychosomatic interventions might have been effective.

According to the Japanese Clinical Guideline for the Diagnosis and Treatment of Interstitial Cystitis/BPS [1] is a condition with hypersensitive bladder symptoms such as bladder pain or urinary frequency in the absence of Hunner lesions and any confusable diseases. BPS is considered associated with the interaction of multiple factors, including neurogenic inflammation, exogenous substances, urothelial defects, psychological stress, and neural hyperactivity. Unfortunately, very few effective therapeutic options are currently available for BPS. Bladder hydrodistension is effective in a certain number of patients with BPS, and its detailed mechanism of action is not known. Previously, we demonstrated that the response rate of hydrodistension for BPS was 50%, and 2 years after hydrodistension, the symptom‐free rate was 63% [3]. Although the patient was diagnosed with BPS without Hunner lesions, the bladder mucosa was fulgurated or resected several times early in the diagnosis because of concerns about missing Hunner lesions due to refractory bladder pain. Chennamsetty et al. reported that bladder capacity did not significantly decrease even with multiple fulgurations [4]. However, in our experience, multiple fulgurations and resections may decrease bladder capacity [3]. The course of this case (Figure 1) appears to indicate that the fulguration and resection performed early in the diagnosis triggered decreases in bladder capacity.

Alexithymia is described as a deficit in the cognitive processing of emotions [5]. Individuals with alexithymia have difficulties in identifying feelings and distinguishing between feelings and bodily sensations that accompany states of emotional arousal. Alexithymia can be diagnosed on the Toronto Alexithymia Scale [6]. In the present patient, alexithymia was diagnosed based on the interview with a palliative care specialist trained in the field of psychiatry. They often avoid social situations, appear cold, show a lack of intimacy and warmth, are insecurely attached to others, and have an externally oriented style of thinking. Previous studies have reported that alexithymia is associated with irritable bowel syndrome, fibromyalgia, tension headache, or temporomandibular disorder, which are considered FSS [7, 8, 9, 10]. Kambara et al. suggested FSS as (1) the main complaint is physical symptoms that are larger than medically explainable, (2) the main symptoms have persisted for more than 6 months, and (3) the symptoms interfere with social activities and daily life [11]. In a review paper of FSS and BPS by Warren et al., they concluded that patients with FSS are at risk for BPS and may benefit from future preventive strategies [12]. Chronic exposure to intense stress in patients with alexithymia and other psychological traits is thought to cause central sensitization, inducing hypersensitivity to pain in FSS [13, 14, 15]. In a report on the involvement of alexithymia in BPS, Chiu et al. advocated that a psychological mechanism independent of a bladder‐centric defect may underlie the mental and somatic symptoms of a subgroup of patients with BPS [16].

Personality traits such as alexithymia cannot be easily changed; thus, affected patients tend to be constantly exposed to psychological stress [5]. They are less likely to notice psychosomatic correlations and more likely to fixate on physical symptoms. In the early stages of the diagnosis of this case, we also recommended psychosocial support intervention; however, the patient's strong refusal made psychosocial interventions difficult. In addition, we could not definitively determine a more effective alternative treatment. Thus, fulguration or resection of the bladder without Hunner lesions must be avoided. We will continue to monitor the patient's bladder capacity, renal function, and presence of hydronephrosis.

## 4 Conclusions

BPS is a heterogeneous group of disorders with a psychosocial background, which may manifest as FSS. Psychosocial and mental health interventions without surgical treatment for the bladder may be persistently attempted.

## Consent

Written informed consent was obtained from the patient.

## Conflicts of Interest

The authors declare no conflicts of interest.
